# Increased Expression of Sox9 during Balance of BMSCs/Chondrocyte Bricks in Platelet-Rich Plasma Promotes Construction of a Stable 3-D Chondrogenesis Microenvironment for BMSCs

**DOI:** 10.1155/2020/5492059

**Published:** 2020-05-26

**Authors:** Ruikai Ba, Liang Kong, Guofeng Wu, Shiyu Liu, Yan Dong, Bei Li, Yimin Zhao

**Affiliations:** ^1^State Key Laboratory of Military Stomatology, Department of Prosthodontics, School of Stomatology, The Fourth Military Medical University, Changle West Road 145, Xi'an, China; ^2^State Key Laboratory of Military Stomatology, Department of Maxillofacial Surgery, School of Stomatology, The Fourth Military Medical University, Changle West Road 145, Xi'an, China; ^3^Department of Prosthodontics, School of Stomatology, Nanjing University, Zhongyang Road, 30 Nanjing, China; ^4^State Key Laboratory of Military Stomatology, Center for Tissue Engineering, School of Stomatology, The Fourth Military Medical University, Changle West Road 145, Xi'an, China

## Abstract

Sox9 is an intrinsic transcription factor related to the determination and maintenance of chondrogenic lineage of bone marrow mesenchymal stem cells (BMSCs). In recent research, we have proved that fragmented chondrocyte aggregates (cell bricks) could promote chondrogenesis of BMSCs in vivo. However, it is still unknown whether the ratio of BMSCs/chondrocyte bricks has a significant influence on 3-D cartilage regeneration and related molecular mechanism. To address this issue, the current study subcutaneously injected three groups of cell complex with different rabbit BMSCs/chondrocyte bricks' ratios (1 : 2, 1 : 1, and 2 : 1) into nude mice. Gross morphology observation, histological and immunohistochemical assays, biochemical analysis, gene expression analysis, and western blot were used to compare the influence of different BMSCs/chondrocyte bricks' ratios on the properties of tissue-engineered cartilage and explore the related molecular mechanism. The constructs of 1 : 1 BMSCs/chondrocyte bricks, (B1CB1) group resulted in persistent chondrogenesis with appropriate morphology and adequate central nutritional perfusion without ossification. The related mechanism is that increased expression of Sox9 in the B1C1 group promoted chondrogenesis and inhibited the osteogenesis of BMSCs through upregulating Col-II as well as downregulating RUNX2 and downstream of Col-X and Col-I by upregulating Nkx3.2. This study demonstrated that BMSCs/chondrocyte bricks 1:1 should be a suitable ratio and the Sox9-Nkx3.2-RUNX2 pathway was a related mechanism which played an important role in the niche for stable chondrogenesis of BMSCs constructed by chondrocyte bricks and PRP.

## 1. Introduction

Repair of cartilage defects has always been a great challenge in treatment due to the poor regenerative capacity of cartilage in vivo [1]. Implantation with an engineered construct composed of autologous chondrocytes is currently a popular approach [2]. However, the lack of autologous cartilage sources and chondrocyte dedifferentiation after in vitro expansion are major obstacles to the clinical application of chondrocyte-based cartilage tissue engineering [3]. In addition, implantation of a prothesis with a specific shape or the transplantation of autologous cartilage may cause a secondary deformity or result in complications including immune rejection, infections, and extrusion [4].

Mesenchymal stem cells (MSCs) are found in numerous tissues throughout the body and are capable of self-renewal [5] and chondrogenic differentiation [6]. Moreover, MSCs can easily be cultured and expanded in vitro, which makes them a particularly attractive cell source for cartilage repair [7]. Previous studies showed that bone marrow mesenchymal stem cells (BMSCs) are easier to obtain than synovial mesenchymal stem cell (SDSC) and have a higher proliferation rate as well as higher expression of cartilage-specific genes and proteins than adipose mesenchymal stem cells (ADSCs) [8, 9], so BMSCs have been extensively investigated in cartilage regeneration [10]. However, BMSCs are easily differentiated into osteocytes when induced by growth factors such as transforming growth factor-*β*1 (TGF-*β*1), bone morphogenetic protein-2 (BMP-2) or fibroblast growth factor-2 (FGF-2) [11–13]. As a result, the engineered cartilage tissue is ultimately vascularized and ossified in vivo [14, 15].

Coculture of BMSCs and chondrocytes is a promising method since chondrocytes can promote the differentiation of BMSCs into a chondrogenic lineage [16]. Additionally, chondrocytes and the extracellular matrix (ECM) can provide a chondrogenic environment for BMSCs that can suppress hypertrophy at the same time [17]. In addition, the consumption of chondrocytes is reduced by the coculture approach. It is possible to repair large defects by harvesting small cartilage biopsies. Even though chondrons with high-ratio MSCs have succeeded in repairing small-size cartilage defects at chondrogenic sites such as articular cartilage defect, long-term research on the efficacy of coculture for enhancing the committed differentiation of MSCs into the chondrogenic lineage for repairing a large cartilage defect at nonchondrogenic sites is still imperative [18, 19]. This is partly because of the complex, delicate, three-dimensional (3-D) culture system that is necessary for both initial chondrogenesis and maintaining the chondrocyte phenotype.

We used a 3-D microenvironment constructed by chondrocyte aggregates (chondrocyte bricks) and PRP for stable chondrogenesis of BMSCs in vivo [20]. We found that this system effectively promoted chondrogenesis of BMSCs. To solve the problem of limited sources of chondrocytes, we wished to use more BMSCs instead of chondrocytes to construct engineered cartilage. However, ossification occurred occasionally when the proportion of BMSCs was higher than that of chondrocytes after long-term growth in vivo. No studies have shown a favorable ratio for BMSCs and chondrocyte bricks (primary chondrocytes) in vivo, which is important information for further efficient clinical application of tissue-engineered cartilage. To address these issues, we prepared three kinds of cell complexes in PRP with different BMSC/chondrocyte bricks' ratios using previously established methods. Here, we compared the shape retention and the quality of the engineered cartilage that was constructed with different cell mixing ratios of BMSCs and auricular chondrocyte bricks in vivo by assessing histology, glycosaminoglycan (GAG) and collagen content, and related gene expression. In this study, we investigated the influence of the BMSC/chondrocyte bricks' ratio and related molecular mechanism on the stable induction of BMSCs into the chondrogenic lineage.

## 2. Materials and Methods

### 2.1. Cell Isolation

All animal experiments were approved by the Institutional Animal Care and Use Committee of The Fourth Military Medical University, Xi'an, PR China (2015-kq-002). BMSCs and chondrocytes were isolated from 4-week-old male New Zealand rabbits (*n* = 3, purchased from the animal center of The Fourth Military Medical University, Xi'an, China) as described previously [20]. Briefly, after being physically disrupted, the bone marrow was washed out from the fragments of the tibiae and femora with 40 ml Dulbecco's modified Eagle's medium (DMEM; HyClone, Logan City, Utah, USA) and centrifuged at 1,500 rpm for 5 minutes. The supernatant was removed, and the cells were twice washed with phosphate-buffered saline (PBS) and then resuspended in DMEM low glucose supplemented with 10% fetal bovine serum (HyClone), 50 *μ*g/ml penicillin, and 30 *μ*g/ml streptomycin (Amresco, Cleveland, Ohio, USA). Primary cells were seeded in a 75 cm^2^ culture flask at 5 × 10^5^ nucleated cells/cm^2^ in DMEM low glucose supplemented with 10% fetal bovine serum (HyClone), L-glutamine (272 *μ*g/ml; Amresco), ascorbate-2-phosphate (50 *μ*g/ml; Sigma, St. Louis, MO, USA), 50 *μ*g/ml penicillin, and 30 *μ*g/ml streptomycin (Amresco) (medium I) and incubated at 37°C with 5% carbon dioxide. After 3 days, the nonadherent cells were removed during the first medium change. Remaining adherent cells were further cultured with a medium change every 3 days. The adherent cells were incubated until cell clones reached over 80% confluence and then were digested with 0.25% trypsin (HyClone) and subcultured at 1.0 × 10^4^ cells/cm^2^. Passage 1 cells (Supplementary Fig. 1A) were used for further experiments after cell counting and testing by Beckman Vi-Cell XR Cell Counter (Beckman Coulter, America).

Chondrocyte bricks were made from fragmented chondrocyte membranes, which were enhanced in a membrane-forming medium (DMEM high glucose supplemented with 20% fetal bovine serum (HyClone, Logan City, Utah, USA), L-glutamine (272 *μ*g/ml; Amresco, Cleveland, Ohio, USA), ascorbate-2-phosphate (50 *μ*g/ml; Sigma, St. Louis, MO, USA), 50 *μ*g/ml penicillin and, 30 *μ*g/ml streptomycin (Amresco, Cleveland, Ohio, USA)) (medium II) by a homemade cutting system according to previously established methods [20]. Briefly, harvested auricular chondrocytes were counted and tested using the Beckman Vi-Cell XR Cell Counter (Beckman Coulter, America) and were cultured at 5.21 × 10^5^ cells/cm^2^ in six-well plates in medium II (Supplementary Fig. 1B). At day 10, when the solid white membrane of chondrocytes formed, the membranes were fragmented in a homemade cutting system in order to achieve chondrocyte bricks (Supplementary Fig. 1C-E).

### 2.2. Preparation of Constructs and Animal Experiments

Platelet-rich plasma (PRP) was enriched from male rabbit whole blood (*n* = 3, New Zealand white rabbits weighing 2.5 to 3.0 kg, purchased from the animal center of The Fourth Military Medical University, Xi'an, China) by a two-step centrifugation process, as described elsewhere [20]. Briefly, 18 ml whole blood was drawn from the ventricle of each rabbit into two sterile tubes, each containing 1 ml sodium citrate (3.8%) solution as an anticoagulant. The tubes were then spun at 1,800 rpm for 8 minutes in a centrifuge at room temperature, and the blood separated into three phases: platelet-poor plasma (top), PRP (middle), and erythrocytes (bottom) (Supplementary Fig. 1F). The top and middle layers were transferred to new tubes and centrifuged again at 3,600 rpm for 8 minutes. The supernatant plasma was discarded, and the remaining 2 ml plasma containing precipitated platelets were blended evenly and designated PRP. The final platelet concentration was adjusted to 20.9 ± 1.1 × 10^7^/ml. PRP was preserved on ice for further steps.

The care and operative procedure of the mice were performed in accordance with the institutional guidelines of the committee of The Fourth Military Medical University. Eighteen 5-week-old male nude mice were randomly divided into three groups (*n* = 6 in each group, 22 to 26 g in weight, purchased from the animal center of The Fourth Military Medical University, Xi'an, China): the B1CB2 group (BMSCs/chondrocyte bricks' ratio of 1 : 2, BMSCs, 1 × 10^7^; primary chondrocytes, 2 × 10^7^), B1CB1 group (BMSCs/chondrocyte bricks' ratio of 1 : 1, BMSCs, 1.5 × 10^7^; primary chondrocytes, 1.5 × 10^7^), B2CB1 group (BMSCs/chondrocyte bricks' ratio of 2 : 1, BMSCs, 2 × 10^7^; primary chondrocytes, 1 × 10^7^). The mice were acclimated for 1 week before operation and monitored for general appearance, activity, excretion, and weight. In total, 3 × 10^7^ cells at the indicated cell ratio were suspended in 500 *μ*l of PRP and injected subcutaneously via a 16 G needle into a nude mouse after the complex was mixed with 50 *μ*l of a thrombogenic agent (100 U/ml in 100 mg/ml calcium chloride; Villalba, Madrid, Spain Biomedical) (Supplementary Fig. 1H, I).

### 2.3. Gross Morphology

Samples were dissected from the surrounding tissues at 14 weeks postinjection for measurement of wet weights, thicknesses, and volumes, as described previously [20]. Briefly, sample volumes were measured by a water displacement method. The specimen was secured with a suture and then dropped gently into a gauge filled with water; the variance of the water volume before and after specimen immersion was calculated and converted into the sample volume. Before measurement, the specimens were evaluated for contour deformation.

### 2.4. Biomechanical and Biochemical Analyses

The mechanical properties of engineered and normal cartilage were analyzed on a biomechanical testing machine (Instron-5542, Canton, MA), according to those described by Zhang et al. [21]. Briefly, a constant compressive strain rate of 0.5 mm/min was applied until 80% of maximal deformation was achieved and a stress strain curve was generated. The Young's modulus of the tested tissue was calculated based on the slope of the stress strain curve. The Young's modulus of engineered cartilage was compared to that of normal cartilage. The result was showed as Young's modulus ratio.

After mechanical testing, the samples were collected and minced for DNA, GAG, and collagen quantifications. The process of collagen and glycosaminoglycan (GAG) quantification was described previously [20]. Briefly, parts of samples that were minced into 1 mm^3^ pieces were digested with pepsin (1 mg/ml in 0.5 M acetic acid; MP, Santa Ana, California, USA) and papain extraction reagent (Sigma, St. Louis, MO, USA) for collagen and GAG contents. Then, measurements of collagen and GAG concentrations in the supernatant were performed with the Sircol™ Collagen Assay (S1000; Biocolor, Carrickfergus, UK) and Blyscan™ GAG Assay Kit (B1000, Biocolor, Carrickfergus, UK), followed by calculation of collagen and GAG per wet weight of construct from the collagen and GAG standard curve, according to the kit instructions. DNA content was determined using the Quant-iT dsDNA assay kit and Qubit Fluorometer system (Invitrogen) according to the manufacturer's instructions. The final results were showed as the content of collagen and GAG per DNA.

### 2.5. Histological and Immunohistochemical Assays

Samples were cut into 6 *μ*m sections after being fixed in paraformaldehyde and embedded in paraffin. Hematoxylin and eosin (H&E), safranin-O, Masson's trichrome, and Von Kossa staining were performed according to standard procedures. Two-step indirect immunohistochemical staining was performed to investigate the expression of type I, type II, and type X collagen in matrices using a primary anti-collagen type I antibody (mouse anti-rabbit, 1 : 50; Abcam, Cambridge, MA, USA), anti-collagen type II antibody (mouse anti-rabbit, 1 : 50; Acris, Herford, Germany), and anti-collagen type X antibody (mouse anti-rabbit, 1 : 50; Abcam, Cambridge, MA, USA), followed by a horseradish peroxidase-conjugated anti-mouse antibody (1 : 200 in PBS; Santa Cruz, Dallas, Texas, USA) and color development with diaminobenzidine tetrahydrochloride (Santa Cruz, Texas, USA). The sections were counterstained with hematoxylin solution (Mayer's).

### 2.6. RNA Isolation and Real-Time RT-PCR

Total RNA was extracted from different groups and rabbit auricular cartilage by RNAiso Plus (TaKaRa, Shiga, Japan), followed by one-step phenol chloroformisoamyl alcohol extraction, as described by the manufacturer's protocol. Real-time RT-PCR analysis of seven genes—Sox9, collagen- (COL-) I (Col1a1), COL-II (Col2a1), COL-X (Col10a1), Nkx3.2, RUNX2, and Gapdh—was performed using the One-Step SYBR® PrimeScript™ RT-PCR Kit (TaKaRa, Shiga, Japan). The results of real-time RT-PCR were replicated five times and presented as target gene expression normalized first to Gapdh in the same sample (*Δ*Ct) and then to the expression of that target gene measured in rabbit auricular cartilage as a native control (*ΔΔ*Ct). The 2^–*ΔΔ*Ct^ method was used to compare differences in gene expression among the three groups. The rabbit primer sequences designed by TaKaRa and used in this study are presented in Table 1.

### 2.7. Western Blot Analysis

Harvested fresh samples were ground in liquid nitrogen and lysed with mammalian protein extraction reagent (Pierce, Rockford, IL, USA) supplemented with complete protease inhibitor. The Pierce BCA Protein Assay Kit (Pierce) was used to determine the total protein content, which was adjusted to 10 *μ*g/*μ*l. Samples (10 *μ*l) were loaded onto 10-14% SDS polyacrylamide gels (SDS-PAGE) and transferred to a polyvinylidene fluoride (PVDF) membrane (Millipore Corporation, Bedford, MA, USA). The membranes were blocked using 5% skim milk in 0.05% Tris-buffered saline/Tween 20 (TBST) for 1 hour. Then, the membrane was incubated at 4°C overnight with the following primary antibodies: anti-Sox9 (Abcam, Cambridge, MA, USA, 1 : 500) and anti-*β*-actin (Cell Signaling, Danvers, MA, 1 : 1000). After further washes, the chemiluminescence of the blot was processed with the ECL western blotting substrate (Pierce).

### 2.8. Data Analysis

All results are expressed as the mean ± standard deviation. Statistical analyses were performed using SPSS 17.0 software (SPSS, Chicago, IL, USA). Under the premise that the data conforms to normal distribution, one-way ANOVA was used for multiple-group comparisons, followed by Tukey's honestly significant difference test; *P* < 0.05 was considered to indicate statistical significance.

## 3. Results

### 3.1. Influence of the BMSCs/Chondrocyte Bricks' Ratios on the Deformation and Contraction of Newborn Cartilage

**Figure 1:**
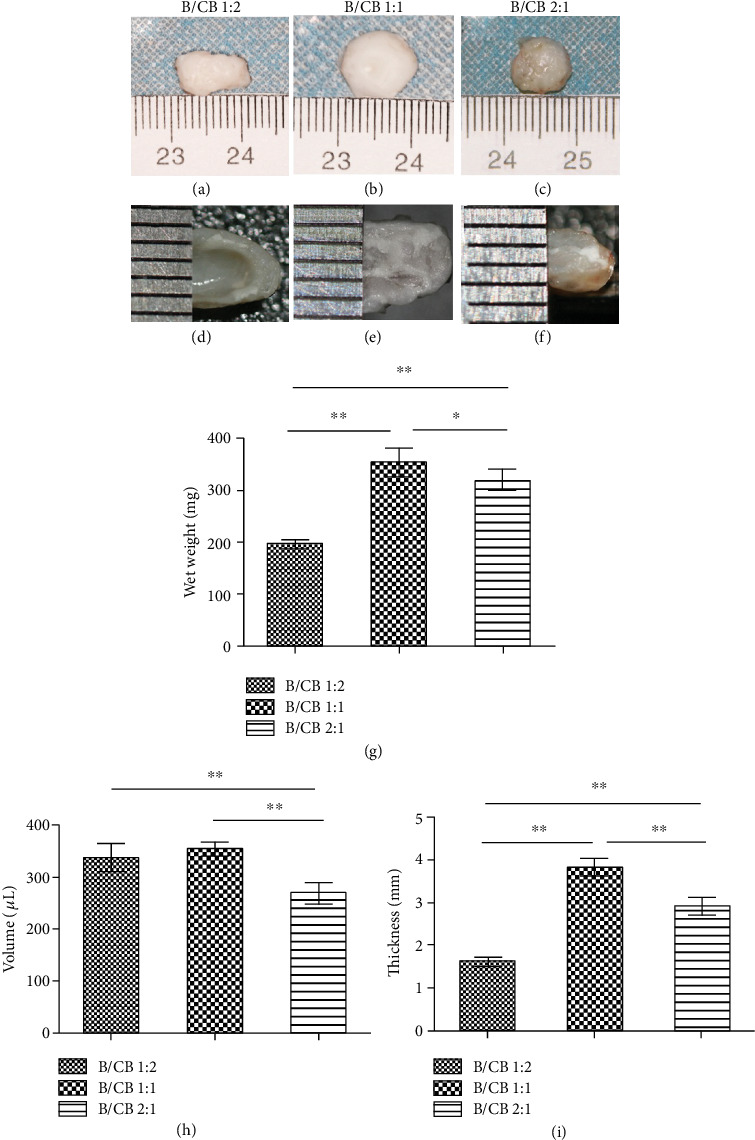
Influence of the BMSCs/chondrocyte bricks' ratio on the deformation and contraction of newborn cartilage. Close macroscopic views of the regenerated cartilage from the B1CB2, B1CB1, and B2CB1 groups after 14 weeks of in vivo incubation. Samples harvested from mice in different groups presented different volumes, weights, and thicknesses. *N* = 6, ^∗∗^*P* < 0.01, ^∗^*P* < 0.05.

In the current study, chondrocyte bricks and PRP were used to provide a chondrogenic niche for chondrogenesis of BMSCs by cotransplantation in the subcutaneous space. As shown in Figure 1, all the specimens in the 1 : 2 BMSCs/chondrocyte bricks' group (B1CB2), 1 : 1 BMSCs/chondrocyte bricks' group (B1CB1), and 2 : 1 BMSCs/chondrocyte bricks' group (B2CB1) formed homogeneous cartilage-like tissues with a white, pearly, opalescent appearance after 14 weeks of subcutaneous implantation (Figures 1(a)‑1(c)). The outline of the B2CB1 group was not as smooth as that of the B1CB1 group. When we observed cross-sections of the samples, we found that necrosis occurred in the central part of the B1CB2 group (Figure 1(d)). To quantitatively evaluate the influence of the BMSCs/chondrocyte bricks' ratios on the deformation and contraction of newborn cartilage, we measured the volumes, thicknesses, and wet weights of regenerated tissues at 14 weeks postoperatively. Although the volumes of the B1CB1 (355.0 ± 12.9 *μ*L) and B1CB2 groups (336.0 ± 28.6 *μ*L) were nearly the same, they were significantly higher than that of the B2CB1 group (268.0 ± 21.1 *μ*l, *P* < 0.01) (Figure 1(h)). As Figure 1(g) shows, the average wet weight of the B1CB1 group (351.8 ± 27.1 mg) was significantly higher than that of the B1CB2 group (195.2 ± 7.2 mg, *P* < 0.01) and the B2CB1 group (316.51 ± 21.62 mg, *P* < 0.05). Accordingly, the thickness measurement of the B1CB1 group (3.8 ± 0.2 mm) was significantly higher than that of the B2CB1 group (2.9 ± 0.2 mm, *P* < 0.01) and the B1CB2 group (1.6 ± 0.1 mm, *P* < 0.01) (Figure 1(i)). All of the above results suggested that a BMSCs/chondrocyte bricks' ratio of 1 : 1 was optimal for maintaining the morphology of newborn cartilage.

### 3.2. Balance of BMSCs/Chondrocyte Bricks Improves Ectopic Chondrogenesis of BMSCs In Vivo

**Figure 2:**
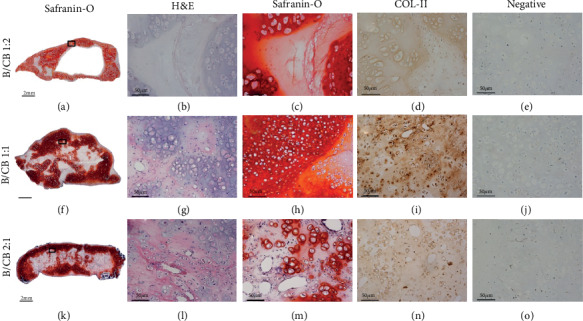
The balance of BMSCs/chondrocyte bricks improves the ectopic chondrogenesis of BMSCs in vivo. Examining chondrogenesis of grafts in vivo at 14 weeks. Merged images showed that the B1CB1 and B2CB1 groups exhibited cell survival and tissue formation throughout the graft (f, k), while the B1CB2 group exhibited central necrosis (a). All three groups presented chondrogenic differentiation of BMSCs, as confirmed by hematoxylin and eosin (H&E), safranin-O staining, and COL-II immunostaining (b‑d, g‑i, l‑n), while osteogenesis occurred outside the samples in the B2CB1 group (l‑n). Scale bar = 2 mm, magnification ×40 (a, f, k); Scale bar = 50 *μ*m, magnification ×200 (b‑e, g‑j, l‑o).

To examine the cartilaginous characteristics of regenerated tissue, histologically, safranin-O staining and type II collagen immunostaining were performed to further reveal remodeling results and the different histological structures of engineered tissues. After 14 weeks in vivo, samples in the B1CB1 and B2CB1 groups presented newborn cartilage tissue formation throughout the grafts, which was in contrast with central necrosis in the B1CB2 group. Cell survival and tissue development could be observed in the interior of the B1CB1 and B2CB1 groups. In the B1CB2 group, regions of round cells with lacunae surrounded by ECM, which is the typical cartilage morphology, were sparsely distributed inside the formed tissues. Deeper safranin-O staining and COL-II immunostaining inside the previous condensing area in the B1CB1 group showed better chondrogenesis of BMSCs than the other two groups (Figure 2(c) and (d), (h) and (i), and (m) and (n)). Moreover, merging of cartilaginous regions and the emergence of cartilage shells were found in the B1CB1 group (Figure 2(f)). Although cartilage shells could also be observed in the B2CB1 group, ossification appeared especially in the outer areas of the sample (Figure 2(k)). In the B1CB2 group, the cartilage shell around the sample was obvious, as the necrosis in the central part (Figure 2(a)).

**Figure 3:**
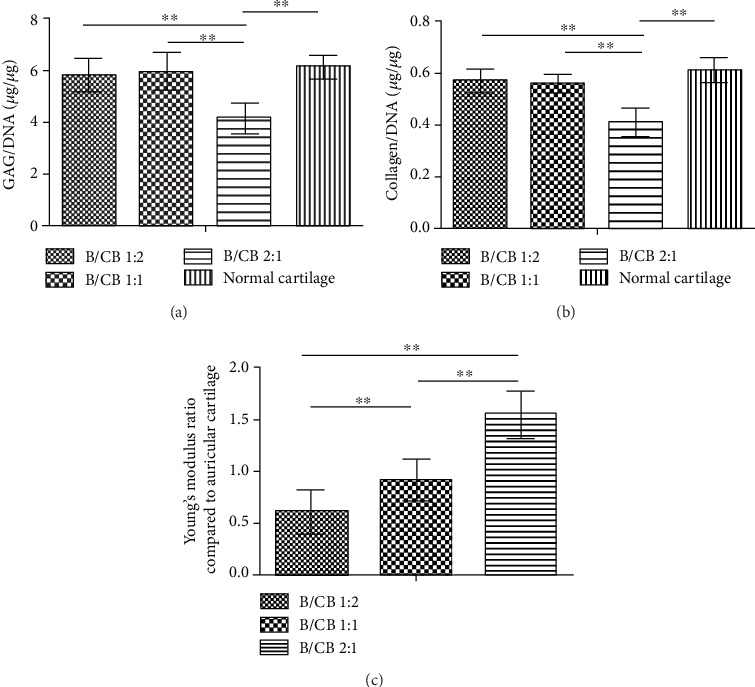
Biochemical and biomechanical characterization. (a, b) Quantitative analysis of the GAG and collagen per DNA at 14 weeks postoperative. (c) Young's modulus ratio compared to auricular cartilage. *N* = 5, ^∗∗^*P* < 0.01.

To further study the influence of the BMSCs/chondrocyte bricks' ratios on chondrogenesis, quantification of collagen and GAG within samples from the three groups was performed. As Figure 3 shows, the GAG content per DNA of the B1CB1 (5.93 ± 0.71 *μ*g/*μ*g) and B1CB2 (5.81 ± 0.61 *μ*g/*μ*g) groups was significantly higher than that of the B2CB1 groups (4.16 ± 0.56 *μ*g/*μ*g, *P* < 0.01). Accordingly, the collagen content per DNA of the B2CB1 group (0.41 ± 0.052 *μ*g/*μ*g) was significantly lower than that of the B1CB1 (0.56 ± 0.032 *μ*g/*μ*g, *P* < 0.01) group and the B1CB2 group (0.57 ± 0.043 *μ*g/*μ*g, *P* < 0.01). What is more, the GAG and collagen content per DNA of B2CB1 was significantly lower than that in normal cartilage (GAG 6.13 ± 0.42 *μ*g/*μ*g, *P* < 0.01; collagen 0.61 ± 0.046 *μ*g/*μ*g, *P* < 0.01) (Figures 3(a) and 3(b)). These results suggested that the promotion of chondrogenesis in the B2CB1 group was not as effective as that in the other two groups. In order to test the mechanical properties of the engineered cartilage for future clinical treatment, we compared Young's moduli of the three groups to normal auricular cartilage after 14 weeks of subcutaneous incubation. Young's modulus of the B1CB1 group reached approximately 92.6% of normal ear cartilage. For the reason of central necrosis, Young's modulus of the B1CB2 groups only reached 61.7% of normal ear cartilage, which was significantly lower than the B1CB1 group (*P* < 0.01). However, Young's modulus of the B2CB1 group (nearly 156.1% of normal ear cartilage) was significantly higher than the B1CB1 and B1CB2 groups (*P* < 0.01) because of the ossification (Figure 3(c)). These results indicated the clinical advantage of the B1CB1 group.

### 3.3. A Higher Proportion of Chondrocyte Bricks Prevents the Ossification of BMSCs at Subcutaneous Nonchondrogenic Sites

**Figure 4:**
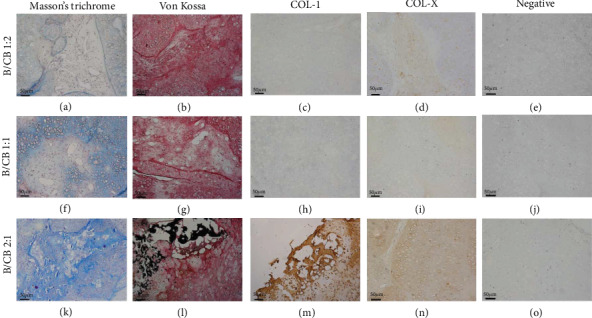
A higher ratio of chondrocyte bricks prevents ossification of BMSCs at subcutaneous nonchondrogenic sites. Examining osteogenesis of grafts in vivo at 14 weeks. In contrast to the B2CB1 group (k‑n), the B1CB1 and B1CB2 groups did not present ossification of BMSCs through 14 weeks (a‑d, f‑i), as confirmed by Von-Kossa, Masson's trichrome, COL-I, and COL-X immunostaining. Scale bar = 50 *μ*m, magnification ×100.

Masson's trichrome staining, Von Kossa staining, and types I and X collagen immunostaining were performed to analyze the influence of the BMSCs/chondrocyte bricks' ratios on the prevention of hypotrophy and ossification of BMSCs at subcutaneous nonchondrogenic sites. Von Kossa staining revealed that calcium, shown as black-colored crystals, was deposited outside the samples, and hypertrophic transition occurred in the B2CB1 group (Figure 4(l)). Masson's trichrome staining of samples demonstrated the production of mature collagen in the B2CB1 group, which confirmed the formation of a mature bone (Figure 4(k)). In contrast, no calcium deposition or cartilaginous collagen production was shown in the B1CB1 and B1CB2 groups, thus presenting chondrogenic performance (Figure 4(a) and (b) and (f) and (g)). To further identify the hypertrophic transition and ossification of implanted BMSCs in different groups, immunostaining for type I and type X collagen was performed. In accordance with histological examination, the newly formed ECM presented deeper staining for type I collagen and type X collagen. Higher expression of the above proteins indicated that BMSCs exposed to the host body in the B2CB1 group underwent hypertrophic transition and ossification (Figure 4(m) and (n)). In contrast, faint staining of both collagens in the B1CB1 and B1CB2 groups indicated that BMSCs in these two groups maintained low-level staining of type I and type X collagen and were protected from ossification at subcutaneous nonchondrogenic sites through 14 weeks (Figure 4(c) and (d) and (h) and (i)).

### 3.4. Higher Expression of Sox9 in the MSC Niche Constructed by Chondrocyte Bricks and PRP Guides Stable Chondrogenesis of BMSCs

**Figure 5:**
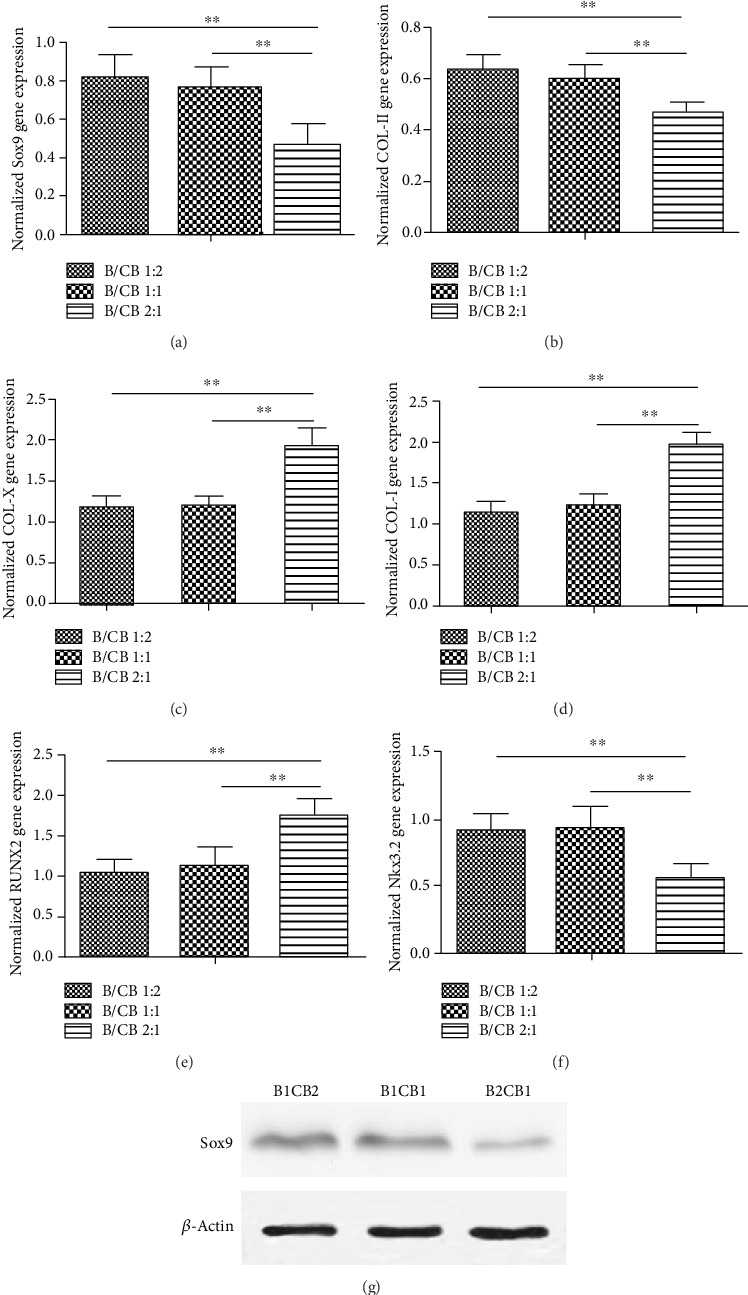
Higher expression of Sox9 in the MSC niche constructed by chondrocyte bricks and PRP guides stable chondrogenesis of BMSCs. Real-time RT-PCR of samples at the 14th week (a‑f) revealed the discrepant expression of Col-I, Col-II, Col-X, RUNX2, Nkx3.2, and Sox9 in different groups, which were all normalized to rabbit auricular cartilage. *N* = 5, ^∗∗^*P* < 0.01. (e) Western blot of Sox9.

We monitored the transcript levels of Col-I, Col-II, and Col-X in cells within the three groups and native cartilage (Figures 5(b) and 5(d)). As shown in Figure 5(b), the expression levels of Col-II genes in the B1CB1 and B1CB2 groups were significantly higher than those in the B2CB1 groups (*P* < 0.01). In contrast, the hypertrophic marker Col-X expression was significantly higher in the B2CB1 group through 14 weeks (Figure 5(c)) (*P* < 0.01). Accordingly, a genetic marker of ossification—Col-I expression—was also higher in the B2CB1 group than that in the other two groups (Figure 5(d)) (*P* < 0.01). These results confirmed histological observations and suggested that chondrocyte bricks played an important role in the microenvironment and that a high ratio of chondrocyte bricks promoted chondrogenesis and prevents ossification of BMSCs.

Moreover, we monitored the expression levels of Sox9. Real-time RT-PCR showed that the transcript levels of Sox9 in the B1CB1 and B1CB2 groups were higher than that of B2CB1 (*P* < 0.01) (Figure 5(a)). The expression level of chondrogenic gene COL-II was significantly higher in the B1CB1 and B1CB2 groups (*P* < 0.01) (Figure 5(b)), while the levels of hypertrophic and osteogenic gene RUNX2 and its downstream genes COL-X and COL-I in the B2CB1 group were significantly higher than those of the other two groups (*P* < 0.01) (Figures 5(c)‑5(e)). What is more, the expression level of Nkx3.2 which acts as a repressor of RUNX2 in the B2CB1 group was significantly lower than that of the B1CB2 and B1CB2 groups (*P* < 0.01) (Figure 5(f)). The results of western blotting also confirmed that the expression level of Sox9 in the B2CB1 group was significantly lower than that in the other two groups (*P* < 0.01) (Figure 5(g)).

## 4. Discussion

One challenge of engineering cartilage with a specific shape is to achieve enough suitable cells for seeding in vivo [16, 21, 22]. Although the limited number of isolated chondrocytes can be sufficiently amplified, the seeded chondrocytes show dedifferentiation, which results in impaired biological and mechanical stability of neocartilage [23]. Recently, coculture of BMSCs and chondrocytes has been widely studied in cartilage tissue engineering [21, 24]. In addition to reducing the consumption of chondrocytes for cartilage construction, it has been demonstrated that cartilage tissue could be generated from BMSCs cocultured with chondrocytes in vitro or in vivo [25, 26]. A further study showed that chondrocyte aggregates (chondrocyte bricks) dispersed uniformly in PRP could provide a chondrogenic niche at a nonchondrogenic site [20], which confirmed that the proliferation and chondrogenic differentiation abilities and stabilities of BMSCs were enhanced by coculture with chondrocyte bricks. It should also be noted that BMSCs could be more homogeneously induced by the chondrogenic microenvironment provided by chondrocyte bricks in coculture systems than by growth factor-mediated chondrogenic induction [27]. More importantly, Fischer et al. [12] found that the parathyroid hormone-related protein secreted by chondrocytes could suppress the hypertrophy induced by BMSCs in a coculture microenvironment.

Nevertheless, ossification occurred occasionally when the proportion of BMSCs was higher than that of chondrocytes bricks after longer term growth in immune animals such as rabbits at a nonchondrogenic site like nasal augmentation in vivo. There have been no systematic comparative studies to determine the effective BMSCs/chondrocyte bricks' ratio for the efficient construction of engineered cartilage in vivo. To avoid interference from external factors, we investigated the influence of three mixed ratios of rabbit BMSCs and chondrocyte bricks (BMSC/original chondrocyte counts of 1 : 2, 1 : 1, and 2 : 1) on chondrogenic and osteogenic differentiation in a PRP construct and compared the shape retention and quality of the engineered cartilage in nude mice. Determination of the shape showed that a 1 : 1 ratio of BMSCs/chondrocyte bricks had a better effect on the stability of the engineered cartilage morphology. The quality of the engineered cartilage was evaluated by assessing the collagen and GAG per DNA in the extracellular matrix as well as Young's modulus ratio compared to normal auricular cartilage. The results of this study showed that a higher ratio of chondrocyte bricks could significantly increase GAG and collagen contents per DNA in the engineered cartilage, which could improve BMSC chondrogenesis and tissue-engineered cartilage formation. In particular, sufficient ECM in chondrocyte bricks provided adequate intrinsic resistance to surrounding pressure and enabled morphological maintenance in a subcutaneous environment. However, a high ratio of chondrocyte bricks that leads to necrosis in the central part of newborn cartilage would inevitably influence its mechanical strength and clinical application. Similarly, ossification which happened in the B2CB1 group also had an adverse impact on future clinical treatment. When we increased the ratio of BMSCs in the complex, an overly high ratio of BMSCs could lead to ossification outside the constructs, which would reduce the elasticity of the constructed cartilage. In this study, we proved that the balance of BMSCs and chondrocyte bricks was important for stable morphology and chondrogenesis as well as prevention of ossification, which provided tissue-engineered cartilage with excellent mechanical property for future clinical treatment.

Previous studies showed that chondrogenically induced MSCs in vitro were apt to calcify with vascular invasion after being implanted subcutaneously [28, 29]. Therefore, the main challenge of MSCs chondrogenesis in the subcutaneous environment is not the failure of chondrogenesis but the failure to maintain the cartilage phenotype. An important finding in this study is that a higher BMSCs/chondrocyte bricks' ratio will reduce the efficiency of protecting BMSCs from ossification at subcutaneous nonchondrogenic sites. In vivo results showed that endochondral ossification started from BMSCs adjacent to the surrounding host tissues. We suspected that a higher BMSCs/chondrocyte bricks' ratio led to the exposure of BMSCs to subcutaneous nonchondrogenic sites caused by fast degradation of peripheral PRP. This opening microenvironment failed to retain paracrine chondrogenic factors released from chondrocytes as well as ECM and protect BMSCs from interference by osteogenic factors, which resulted in progressive ossification of BMSCs. Because of the limitation of nude mouse lifetime, for the further study, we will use a rabbit auricular and articular defect model to test our results.

The effects of the coculture on the cartilaginous and osseous phenotype in the PRP construct in vivo are the result of multiple factors, including growth factors [30]. Interactions of soluble factors from MSCs and chondrocytes as well as PRP play significant roles in multiple steps during chondrogenic and osteogenic differentiation [31]. The decision for MSCs to differentiate depends on two main transcription factors: Sox9 for chondrogenesis and RUNX2 for osteogenesis [32, 33]. Sox9 has a dominant role in promoting chondrogenic differentiation by positively regulating MSC differentiation and represses RUNX2 by Nkx3.2 directly [34, 35]. RUNX2 expression stimulates differentiation of the osteoblast lineage and contributes to chondrocyte development by regulating maturation into hypertrophic chondrocytes [36]. In our study, we found that the expression level of Sox9, RUNX2, Nkx3.2, COL-I, COL-II, and COL-X varied with variations in the BMSCs/chondrocyte bricks' ratios. The expression level of Sox9 significantly increased when the ratio of chondrocyte bricks was high. Moreover, the expression level of chondrogenesis marker gene COL-II was upregulated in the higher ratio of chondrocyte brick groups (B1CB1 and B1CB2). On the contrary, hypertrophy and ossification-related gene RUNX2 was significantly increased in a high ratio in the BMSC group, and its downstream gene COL-X and COL-I expression was upregulated in the B2CB1group. At the same time, the expression levels of upstream gene Nkx3.2 in the B1CB1 and B1CB2 groups were significantly upregulated. As with the histological results related to the prevention of ossification, the expression level of Sox9 increased with the increasing of the chondrocyte bricks ratio, showing that the Sox9-Nkx3.2-RUNX2 pathway played an important role in the niche for chondrogenesis of BMSCs constructed by chondrocyte bricks and PRP. With regard to the limitation of cell species, studies of the interactions of Sox9 with other growth factors in human cells are still underway.

## 5. Conclusions

The study demonstrated that the balance of BMSCs and chondrocyte bricks played a leading role in maintaining stable chondrogenesis and inhibiting ossification of BMSCs in PRP constructs after subcutaneous injection in nude mice and that a 1 : 1 BMSC/chondrocyte bricks' ratio should be a relatively suitable ratio for cartilage regeneration in vivo. Moreover, the increased expression of Sox9 associated with the balance of BMSCs/chondrocyte bricks was a related factor in maintaining a chondrogenic lineage of BMSCs in the 3-D chondrogenesis microenvironment we constructed.

## Figures and Tables

**Table 1:** Gene primer sequence for real time-PCR.

Genes	Primers
*Col1a1*	Forward 5′-GACATGTTCAGCTTTGTGGACCTC-3′
	Reverse 5′-GGGACCCTTAGGCCATTGTGTA-3′
*Col2a1*	Forward 5′-GACCCCATGCAGTACATG-3′
	Reverse 5′-GACGGTCTTGCCCCACTT-3′
*Col10a1*	Forward 5′-GGGATGCCTCTTGTCAGTGC-3′
	Reverse 5′-ATCTTGGGTCATAGTGCTGCTG-3′
*Sox9*	Forward 5′-AATCTCCTGGACCCCTTCAT-3′
	Reverse 5′-GTCCTCCTCGCTCTCCTTCT-3′
*Runx2*	Forward 5′-GACTGTGGTTACCGTCATGG-3′
	Reverse 5′-ACTTGGTTTTTCATAACAGC-3′
*Nkx3.2*	Forward 5′-GATGCGGGCCGCCAAGGACC-3′
	Reverse 5′-AGGAGGCGGGAGCCCGACAC-3′
*Gapdh*	Forward 5′-TGGTATCGTGGAAGGACTCATGAC-3′
	Reverse 5′-ATGCCAGTGACGTTCCCGTTCAGC-3′

## Data Availability

The data used to support the findings of this study are included within the article.

## Supplementary Materials

Supplementary Materials Supplementary Figure 1: preparation of constructs and animal experiments: (A) BMSCs. (B) Chondrocytes. (C-E) Process of achieving chondrocyte bricks. (F) Process of making PRP. (G) Constructs of cell complex with PRP before transplantation. (H-I) Subcutaneous injection.
