# Supplementary material for: Increased Expression of Sox9 during Balance of BMSCs/Chondrocyte Bricks in Platelet-Rich Plasma Promotes Construction of a Stable 3-D Chondrogenesis Microenvironment for BMSCs
Source: Stem Cells Int. 2020 May 26;2020:5492059. doi: 10.1155/2020/5492059 (PMC7271054; doi:10.1155/2020/5492059)
Supplement: Supplementary Materials — Supplementary Figure 1: preparation of constructs and animal experiments: (A) BMSCs. (B) Chondrocytes. (C-E) Process of achieving chondrocyte bricks. (F) Process of making PRP. (G) Constructs of cell complex with PRP before transplantation. (H-I) Subcutaneous injection. [file 5492059.f1.pdf]

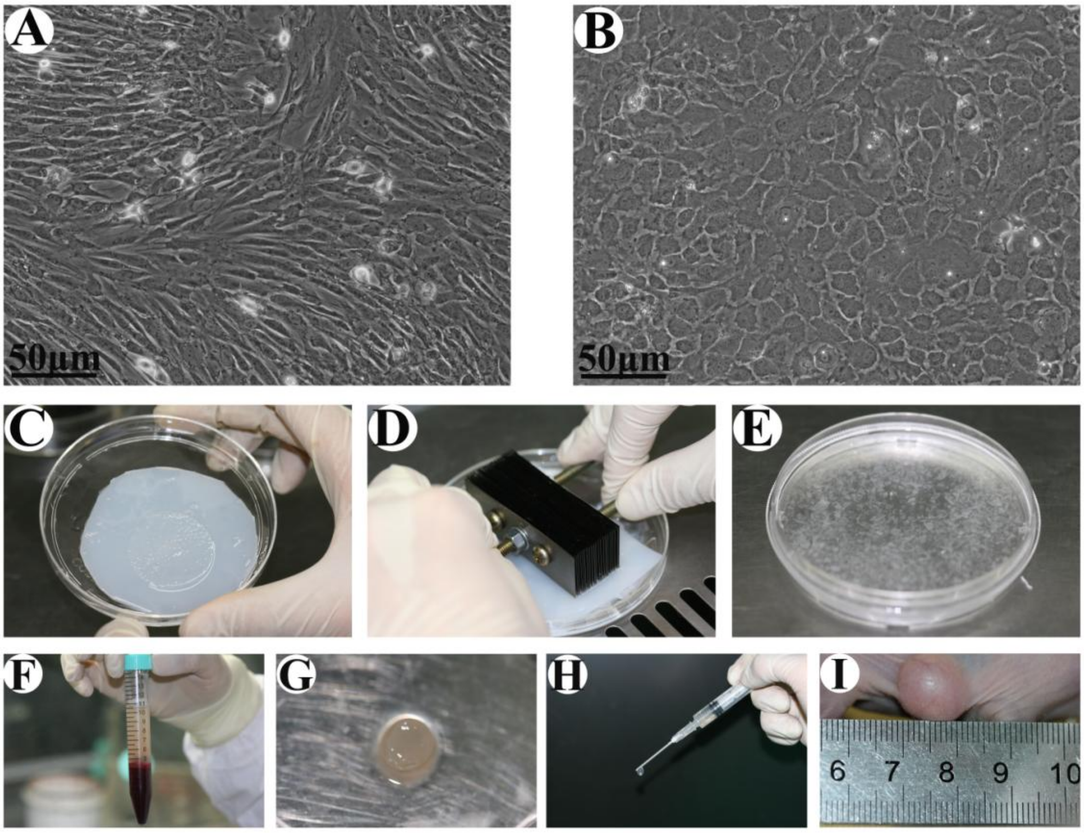

Supplementary Fig.1. **Preparation of constructs and animal experiments: (A)** BMSCs. **(B)** Chondrocytes. (C-E) Process of achieving chondrocyte bricks. (F) Process of making PRP. (G) Constructs of cell complex with PRP before transplantation. (H-I) Subcutaneous injection.
